# Cr(II) and Cr(III) NCN pincer complexes: synthesis, structure, and catalytic reactivity

**DOI:** 10.1007/s00706-023-03128-6

**Published:** 2023-10-10

**Authors:** Matthias G. Käfer, Wolfgang Eder, Jan Pecak, Berthold Stöger, Marc Pignitter, Luis F. Veiros, Karl Kirchner

**Affiliations:** 1 Institute of Applied Synthetic Chemistry, TU Wien, Getreidemarkt 9/163-AC, 1060 Vienna, Austria; 2 X-Ray Center, TU Wien, Getreidemarkt 9/163-AC, 1060 Vienna, Austria; 3 Department of Physiological Chemistry, Faculty of Chemistry, University of Vienna, Althanstraße 14, 1090 Vienna, Austria; 4 Centro de Química Estrutural, Institute of Molecular Sciences, Departamento de Engenharia Química, Instituto Superior Técnico, Universidade de Lisboa, Av. Rovisco Pais, 1049 001 Lisbon, Portugal

**Keywords:** Pincer complexes, Chromium, Hydrosilylation, Silanes, Ketones

## Abstract

The synthesis, characterization, and reactivity of several new Cr(II) and Cr(III) complexes featuring an NCN pincer ligand with an arene backbone connected to amine donors NEt_2_ and N*i*Pr_2_ via CH_2_-linkers is described. Reacting the in situ lithiated ligand precursor N(C–Br)N^CH2^-Et with [CrCl_3_(THF)_3_] resulted in the formation of the Cr(III) complex *trans*-[Cr(κ^3^*NCN*-NCN^CH2^-Et)(Cl)_2_(THF)]. Upon reaction of lithiated N(C–Br)N^CH2^-*i*Pr with a suspension of anhydrous CrCl_2_, the Cr(II) complex [Cr(κ^2^*NC*-NCN^CH2^-*i*Pr)_2_] is formed featuring two NCN ligands bound in κ^2^*NC*-fashion. In contrast, when lithiated N(C–Br)N^CH2^-*i*Pr is reacted with a homogeneous solution of anhydrous Cr*X*_2_ (*X* = Cl, Br), complexes [Cr(κ^3^*NCN-*NCN^CH2^-*i*Pr)X] are obtained. Treatment of [Cr(κ^3^*NCN-*NCN^CH2^-*i*Pr)Cl] with 1 equiv of PhCH_2_MgCl and LiCH_2_SiMe_3_ afforded the alkyl complexes [Cr(κ^3^*NCN*-NCN^CH2^-*i*Pr)(CH_2_Ph)] and [Cr(κ^3^*NCN*-NCN^CH2^-*i*Pr)(CH_2_SiMe_3_)]. All Cr(II) complexes exhibit effective magnetic moments in the range of 4.7–4.9 *µ*_B_ which is indicative for *d*^4^ high spin systems. If a solution of lithiated N(C–Br)N^CH2^-*i*Pr is treated with CrCl_2_, followed by addition of an excess of Na[HB(Et)_3_], the dimeric complex [Cr(κ^2^*NC*-NCN^CH2^-*i*Pr)(μ_2_-H)]_2_ is obtained bearing two bridging hydride ligands. [Cr(κ^3^*NCN*-NCN^CH2^-*i*Pr)(CH_2_SiMe_3_)] turned out to be catalytically active for the hydrosilylation of ketones at room temperature with a catalyst loading of 1 mol%. X-ray structures of all complexes are presented.

**Graphical abstract:**

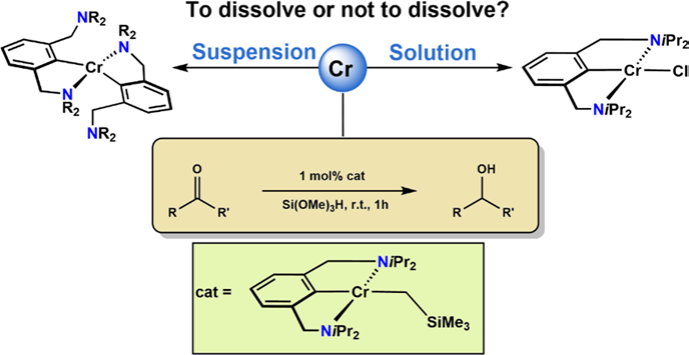

**Supplementary Information:**

The online version contains supplementary material available at 10.1007/s00706-023-03128-6.

## Introduction

In contrast to the chemistry of transition metal PCP pincer complexes [1–12] which feature an aromatic anionic arene backbone connected to phosphine donors via various linkers (CH_2_, O, NH, NR), the chemistry of NCN pincer complexes featuring amine donors instead is rich but largely limited to Ni, Pd, and Pt [13–19]. Most notably, van Koten and coworkers prepared numerous Pd and Pt complexes for applications in catalysis, sensor systems, or even as building blocks for biomolecular and peptide chemistry. The major difference affecting the coordination chemistry of NCN ligands is that the N-atom is significantly smaller than the corresponding P-atom in PCP ligands and the aliphatic NR_2_ group acts exclusively as a σ-donor. Moreover, NCN ligands are coordinated typically in planar tridentate *mer*-fashion, but in some cases also a *fac* geometry was observed [19].

Expanding on previous work with cobalt NCN pincer complexes [20] we envisioned a further contribution to the virtually non-existing field of chromium NCN pincer chemistry. It has to be noted that some Cr(III), Cr(II), and Cr(I)) PCP pincer complexes were reported recently [21–23]. Moreover, Cr(II) complexes containing monoanionic PNP and NNN-pincer type ligands featuring a pyrrole backbone were reported by Gade and coworkers (Scheme 1) [24, 25]. Gao, et al. [26] described the synthesis of Cr(III) bis(imino)aryl NCN pincer complexes which were applied as catalysts for isoprene polymerization.

**Figure:**
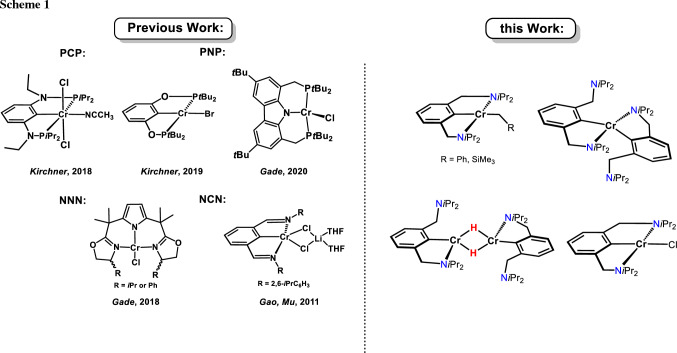

Herein we report on the synthesis, characterization, and reactivity of several new Cr(II) and Cr(III) complexes featuring an NCN pincer-ligand with an arene backbone connected to amine donors NEt_2_ and N*i*Pr_2_ via CH_2_-linkers. Representative X-ray structures, EPR-spectra, and DFT calculations are presented.

## Results and discussion

**Fig. 1:**
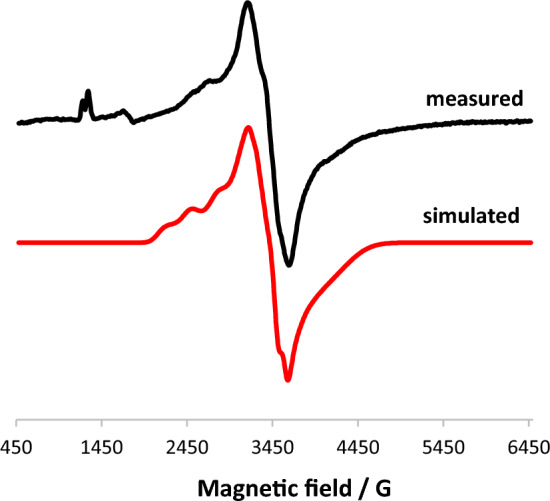
X-band EPR spectrum at a microwave frequency of 9.86 GHz of [Cr(κ^3^*NCN*-NCN^CH2^-Et)(Cl)_2_(THF)] (**3**) in frozen toluene glass at 100 K

The in situ lithiation of N(C–Br)N^CH2^-Et (**1**) with *n*-BuLi in THF at − 90 °C followed by addition of [CrCl_3_(THF)_3_] resulted, after workup, in the formation of complex *trans*-[Cr(κ^3^*NCN*-NCN^CH2^-Et)(Cl)_2_(THF)] (**3**) in isolated 63% yield (Scheme 2). The measurement of the solution magnetic properties (Evans method, benzene [27]) revealed an effective magnetic moment of 3.7(2) *μ*_B_ which is consistent with three unpaired electrons as expected for a *d*^3^ configuration and an oxidation state of + III. Figure 1 depicts the EPR spectrum of **3** obtained from a frozen toluene solution at 100 K, which confirms the expected quartet ground state. This spectrum has been successfully simulated using the following parameters: *g*_*x*_ = 1.989, *g*_*y*_ = 1.764, *g*_*z*_ 2.392 (*g*_iso_ = 2.048) and ^53^Cr hyperfine coupling constants *A*_*x*,1_ = 82.575, *A*_*y*,1_ = 0.009, *A*_*z*,1_ = 315.588 and *A*_*x*,2_ = 19.677, *A*_*y*,2_ = 251.114 and *A*_*z*,2_ = 107.022. The observed effective *g*_iso_-value is typical for an electronic spin of *S* = 3/2.

**Figure:**
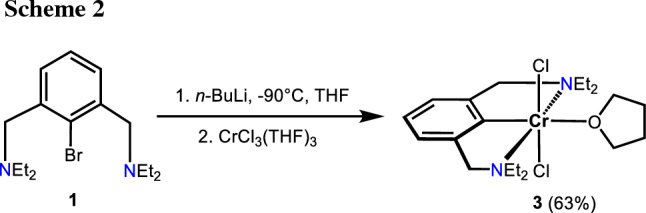

**Fig. 2:**
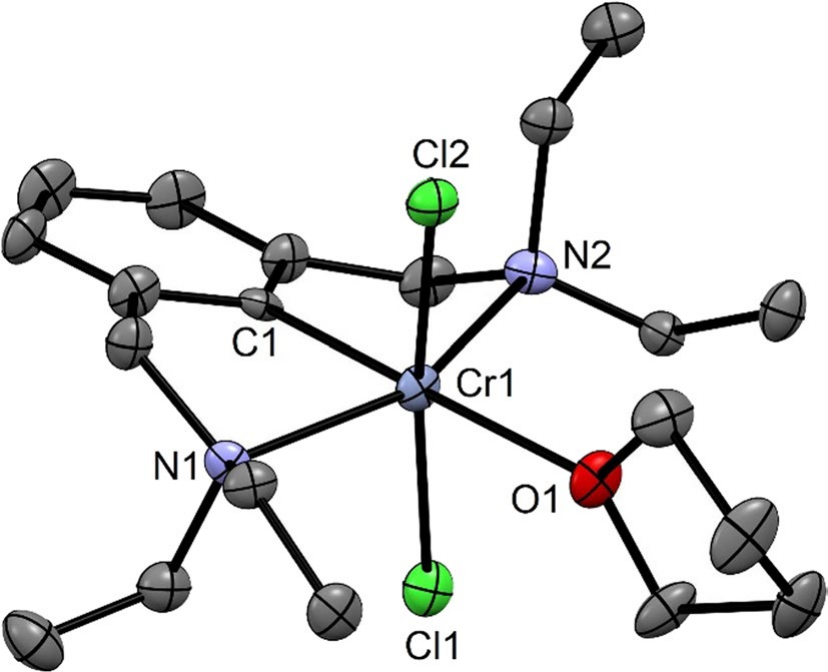
Structural views of *trans*-[Cr(κ^3^*NCN*-NCN^CH2^-Et)(Cl)_2_(THF)] (**3**) showing 50% thermal ellipsoids (H atoms are omitted for clarity). Selected bond lengths (Å) and bond angles (°): Cr1-C1 1.995(7), Cr1-N1 2.294(7), Cr1-N2 2.293(7), Cr1-Cl1 2.377(3), Cr1-Cl2 2.377(3), Cr1-O1 2.245(6), C20-Cr1-O1 179.5(4), N2-Cr1-N1 155.1(3), Cl2-Cr1-Cl1 171.82(9)

To unequivocally establish the ligand arrangement and geometry, single crystals were grown by slow diffusion of *n*-pentane into a saturated THF at room temperature. A view of the molecular structure is depicted in Fig. 2 with selected metrical parameters reported in captions. The complex adopts a distorted octahedral geometry. While the C1–Cr1–O1 and Cl1–Cr1–Cl2 angles deviate only slightly from linearity being 179.5(4)° and 171.5(4)°, respectively, whereas the N1–Cr1–N2 angle deviate significantly from 180° being 155.1(3)°. Similar geometries were recently found for the analogous complexes *trans*-[Cr(κ^3^*PCP-*POCOP-*i*Pr)(Br)_2_(THF)], *trans*-[Cr(κ^3^*PCP*-PCP^NEt^-*i*Pr)(Cl)_2_(THF)], and *trans*-[Cr(κ^3^*PCP*-PCP^CH2^-*i*Pr)(Br)_2_(CH_3_CN)] [21, 23]. The Cr–C_ipso_ distance is 1.995(7) Å being comparable to those of other Cr(III) PCP pincer complexes. The Cr–C_*ipso*_ bond distance in [Cr(κ^3^*PCP*-POCOP-*t*Bu)(Br)], *trans*-[Cr(κ^3^*PCP*-POCOP-*i*Pr)(Br)_2_(THF)], [Cr(κ^3^*PCP*-POCOP-*t*Bu)(κ^2^-BH_4_)], [Cr(κ^3^*PCP*-POCOP-*t*Bu)(NO)(κ^2^-BH_4_)], [Cr(κ^3^*PCP*-POCOP-*t*Bu)(NO)(Br)] [22], and *trans*-[Cr(κ^3^*PCP*-PCP^CH2^-*i*Pr)(Br)_2_(CH_3_CN)] [23] are 2.084(3), 2.067(5), 2.076(2), 2.068(3), 2.056(2), and 2.052(1) Å, respectively.

Treatment of lithiated N(C–Br)N^CH2^-Et (**1**) with anhydrous CrCl_2_ resulted in the formation of intractable paramagnetic products. Gratifyingly, the reaction of the bulkier ligand N(C–Br)N^CH2^-*i*Pr (**2**) with a suspension of anhydrous CrCl_2_ under otherwise similar reaction conditions afforded, after workup, [Cr(κ^2^*NC*-NCN^CH2^-*i*Pr)_2_] (**4**) in 37% isolated yield (Scheme 3). This complex contains two NCN ligands bound in κ^2^*NC*-fashion. Evaluation of the solution magnetic properties of **4** (Evans method, benzene) showed an effective magnetic moment of 4.7(3) *µ*_B_ which is indicative for a *d*^4^ high spin system featuring four unpaired electrons.

**Figure:**
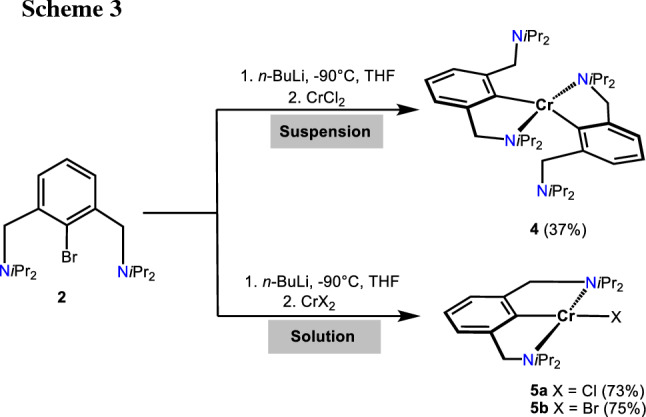

**Fig. 3:**
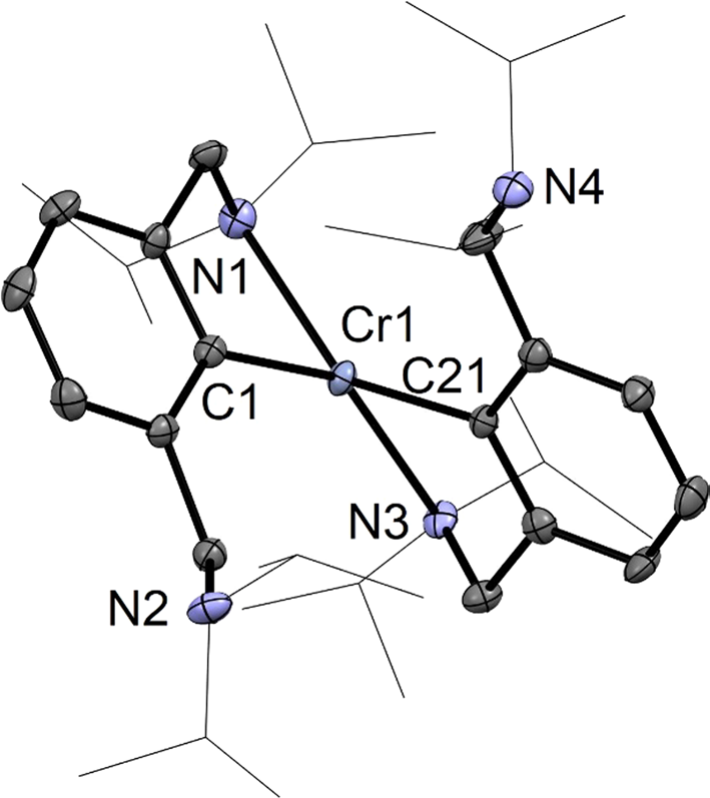
Crystal structure of [Cr(κ^2^*NC*-NCN^CH2^-*i*Pr)_2_] (**4**) with 50% thermal ellipsoids (H-atoms omitted for clarity). Selected bond lengths (Å) and bond angles (°): Cr1-C1 2.073(4), Cr-C21 2.090(4), Cr1-N1 2.381(4), Cr-N3 2.371(4), C1-Cr1-N3 145.0(1), C21-Cr1-N1 143.7(2)

Cooling of a saturated *n*-pentane solution of **4** to − 30 °C led to the formation of crystals suitable for single-crystal X-ray diffraction studies. A structural view of **4** is depicted in Fig. 3 with selected bond distances and angles given in the caption. Complex **4** adopts a strongly distorted tetrahedral coordination geometry as also seen from the structural parameters τ_4_ and τ_4_′  being 0.51 and 0.50, respectively (*τ*_4_ = *τ*_4_′ = 0 indicates an ideal square planar structure, *τ*_4_ = *τ*_4_′ = 1 indicates an ideal tetrahedral structure and a *τ*_4_ ≈ 0.43 and *τ*_4_′ ≈ 0.24 represents a seesaw geometry) [28]. The NCN ligand is coordinated in κ^2^*NC*-fashion and features one pendant amine arm.

In contrast, when the lithiated ligand N(C–Br)N^CH2^-*i*Pr (**2**) is reacted with a solution rather than a suspension of anhydrous CrCl_2_ or CrBr_2_ in THF, obtained after ultrasonic irradiation, complexes [Cr(κ^3^*NCN-*NCN^CH2^-*i*Pr)Cl] (**5a**) and [Cr(κ^3^*NCN-*NCN^CH2^-*i*Pr)Br] (**5b**) were obtained in 73 and 75% isolated yields (Scheme 3). As indicated by solution magnetic susceptibility measurements (Evans method, benzene), these compounds are high-spin complexes with a solution-effective magnetic moment of 4.8(2) and 4.7(2) *μ*_B_. This is in agreement with a high-spin *d*^4^ center (four unpaired electron) and is in the same range as the theoretical spin-only value of 4.90 *μ*_B_.

**Fig. 4:**
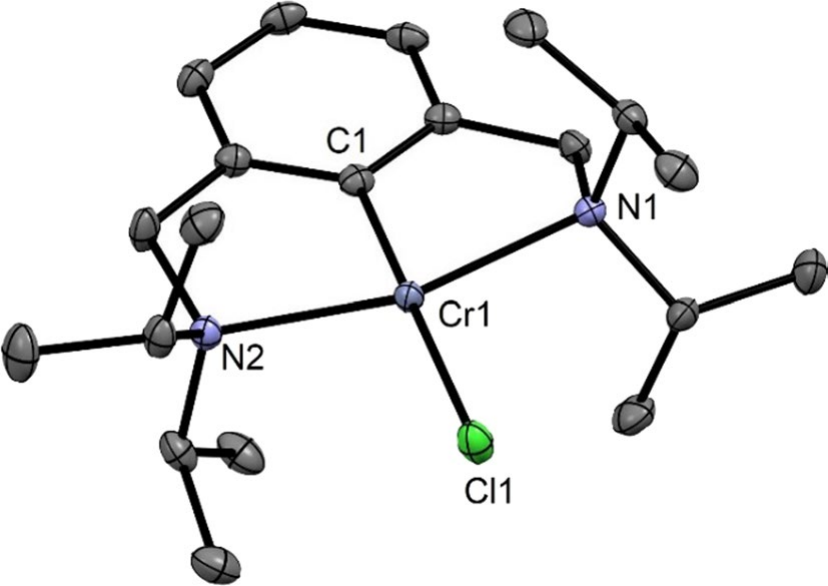
Crystal structure of [Cr(κ^3^*NCN-*NCN^CH2^-*i*Pr)Cl] (**5a**) with 50% thermal ellipsoids (H-atoms omitted for clarity). Selected bond lengths (Å) and bond angles (°): Cr1-C1 2.011(2), Cr1-N1 2.261(2), Cr1-N2 2.257(2), Cr-Cl1 2.4546(6), C1-Cr1-Cl1 178.59(7), N1-Cr1-N2 158.52(8)

The molecular structure of **5a** shows the metal in a typical distorted-square planar configuration (*τ*_4_ = 0.16, *τ*_4_′ = 0.10) [27]. The C1–Cr1–Cl1 angle deviates slightly from linearity 178.59(7)°. The N1–Cr1–N2 angle is 158.52(8)°. The Cr–C_*ipso*_ bond distance of 2.011(2) Å is comparable to those of other Cr(II) pincer complexes [22] (Fig. 4)

**Fig. 5:**
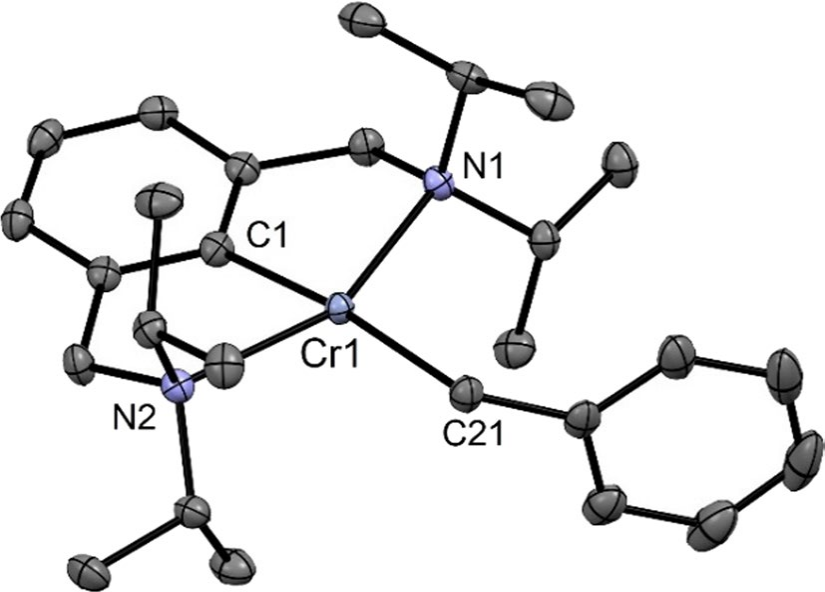
Crystal structure of [Cr(κ^3^*NCN-*NCN^CH2^-*i*Pr)(CH_2_Ph)] (**6**) with 50% thermal ellipsoids (H-atoms omitted for clarity). Selected bond lengths (Å) and bond angles (°): Cr1-C1 2.043(1), Cr1-N1 2.291(1), Cr1-N2 2.327(1), Cr1-C21 2.249(1), C1-Cr1-C21 173.29(5), N1-Cr1-N2 154.99(4)

Treatment of **5a** with 1 equiv of PhCH_2_MgCl and LiCH_2_SiMe_3_ in toluene for 1.5 h afforded the alkyl complexes [Cr(κ^3^*NCN*-NCN^CH2^-*i*Pr)(CH_2_Ph)] (**6**) and [Cr(κ^3^*NCN*-NCN^CH2^-*i*Pr)(CH_2_SiMe_3_)] (**7**), respectively, in 72 and 68% isolated yields (Scheme 4). It has to be noted that complexes **6** and **7** can also be obtained directly from **2** via a one-pot reaction (see “Experimental”). Solution magnetic susceptibility measurements (Evans method, benzene) show that these complexes are also *d*^4^-high-spin complexes with effective magnetic moments of 4.9(1) and 4.8(1) μ_B_. A structural view of **6** is shown in Fig. 5 with selected bond distances and angles reported in the caption. The coordination geometry around the chromium center is best described by a slightly distorted square-planar arrangement with *τ*_4_ and *τ*_4_′ values of 0.22 and 0.17, respectively. The C1–Cr1–C21 angle deviates from linearity being 173.29(5)°. The N1–Cr1–N2 angle is 154.99(4)°. The Cr–C_*ipso*_ and the Cr–C_alkyl_ bond distances are 2.043(1) and 2.249(1) Å, respectively.

**Figure:**
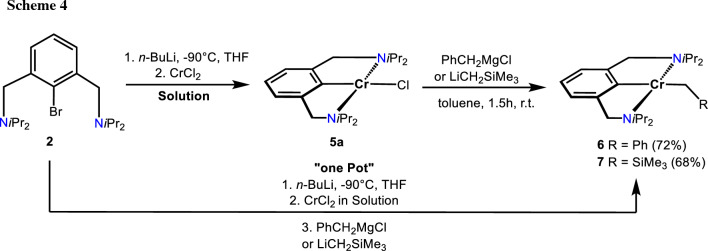

If a solution of lithiated N(C–Br)N^CH2^-*i*Pr (**2**) is treated with anhydrous CrCl_2_, followed by the addition of an excess of Na[HB(Et)_3_] (3.5 equiv, 1 M solution in THF) at − 30 °C, after work up, the dimeric complex [Cr(κ^2^*NC*-NCN^CH2^-*i*Pr)(μ_2_-H)]_2_ (**8**) is obtained in 35% isolated yield (Scheme 5). A solution magnetic moment of *μ*_eff_ = 6.9(3) *μ*_B_ (Evans method, benzene) was determined which is consistent with eight unpaired electrons as expected for a high-spin *d*^4^ configuration of the two metal centers. This also suggests that complex **8** contains no metal–metal bond (*vide infra*).

**Figure:**
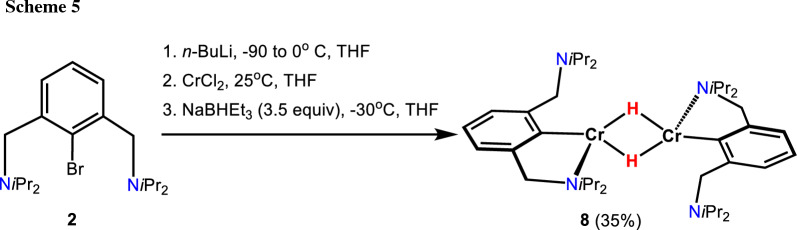

**Fig. 6:**
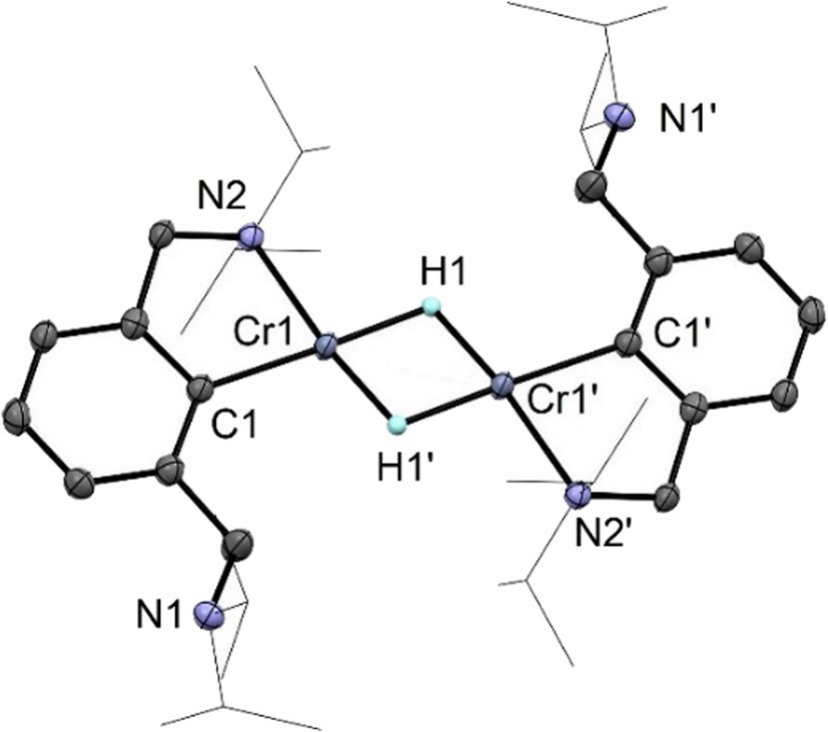
Crystal structure of [Cr(κ^2^*NC*-NCN^CH2^-*i*Pr)(μ_2_-H)]_2_ (**8**) with 50% thermal ellipsoids (most H-atoms omitted for clarity). Selected bond lengths (Å) and bond angles (°): Cr1-C1 2.099(2), Cr1-N1 2.171(1), Cr1-H1 1.78(1), Cr1-Cr1′ 2.6941(5), C1-Cr1-H1 173.2(1), N2-Cr1-H1′ 177.8(1)

The molecular structure of **8** was unequivocally determined by X-ray crystallography. A structural view of **8** is given in Fig. 6 with selected bond distances and angles given in the caption. Complex **8** contains two µ_2_-hydride ligands bridging the two Cr(II) centers. The distance of Cr1 to its image by inversion symmetry is 2.6941(6) Å which is in line with structurally related compounds found in literature [24–26, 29, 30]. Furthermore, **8** adopts an almost perfect square planar geometry around the chromium atoms with *τ*_4_ and *τ*_4_′ values of 0.06 and 0.05, respectively.

**Fig. 7:**
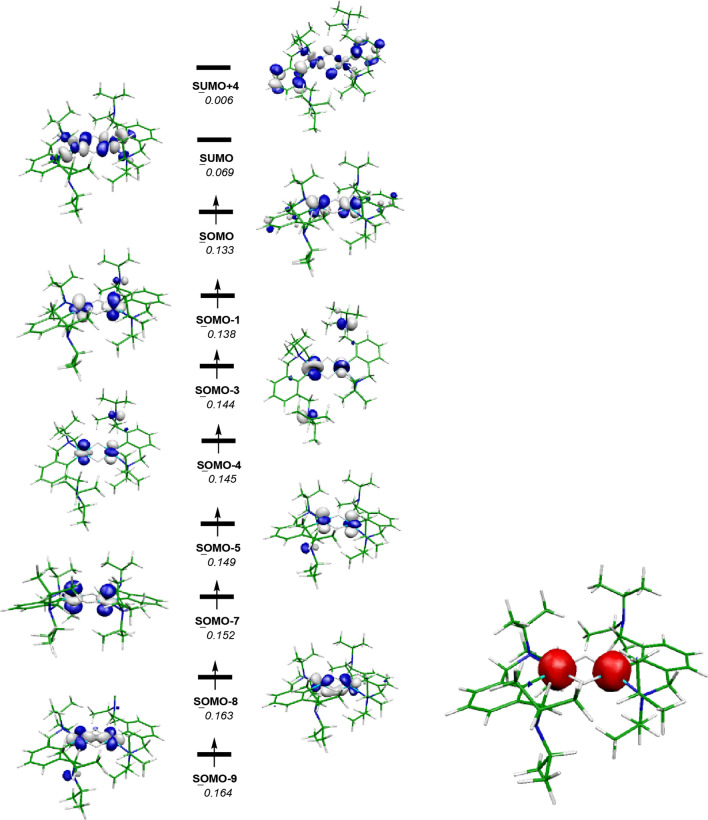
Relevant orbitals (d-splitting, α-spin orbitals) and spin density for [Cr(κ^2^NC-NCN.^CH2^-*i*Pr)(μ_2_-H)]_2_ (**8**). Orbital energy values in hartrees (italics)

The relevant orbitals obtained by DFT calculations for complex **8** are depicted in Fig. 7 and are consistent with two high-spin d^4^ Cr(II) centers. Accordingly, the spin density of the molecule is located on the metal atoms as also shown in Fig. 7. The relevant Wiberg index (WI)^1^ [31] for the Cr⋅⋅⋅Cr interaction is 0.15 indicating essentially no Cr–Cr metal bond. The two µ_2_-hydrogen atoms are of a hydridic nature with NPA charges of − 0.35.

Since a Cr(II) PCP alkyl complex was recently shown to be catalytically active for the hydrosilylation of ketones [22], we investigated the potential of the Cr(II) NCN alkyl complex **7** as a catalyst for this transformation. Complex **7** (1 mol% based on ketones) was reacted with both aromatic and aliphatic ketones and Si(OMe)_3_H (2 equiv) in toluene (3 cm^3^) at 25 °C. After workup with K_2_CO_3_/MeOH, all alcohols were isolated in yields up to 89% and were characterized by ^1^H, ^13^C{^1^H}, and ^19^F{^1^H} NMR spectroscopy. These results are summarized in Table 1. The protocol tolerates halide (**9a**, **9b**), ether (**9c**–**9e**), and thiophene (**9i**) functionalities. Lower or no conversion was observed in the presence of amine (**9h**) and hydroxy (**9l**) groups. Likewise, aromatic ketones with substituents in the ortho positions resulted in no conversion (**9k**, **9m**). In sum, the catalytic activity of **7** is very similar to the analogous Cr(II) PCP pincer complex [Cr(κ^3^*PCP-*POCOP-*i*Pr)-(CH_2_SiMe_3_)] [22].

**Table 1:**
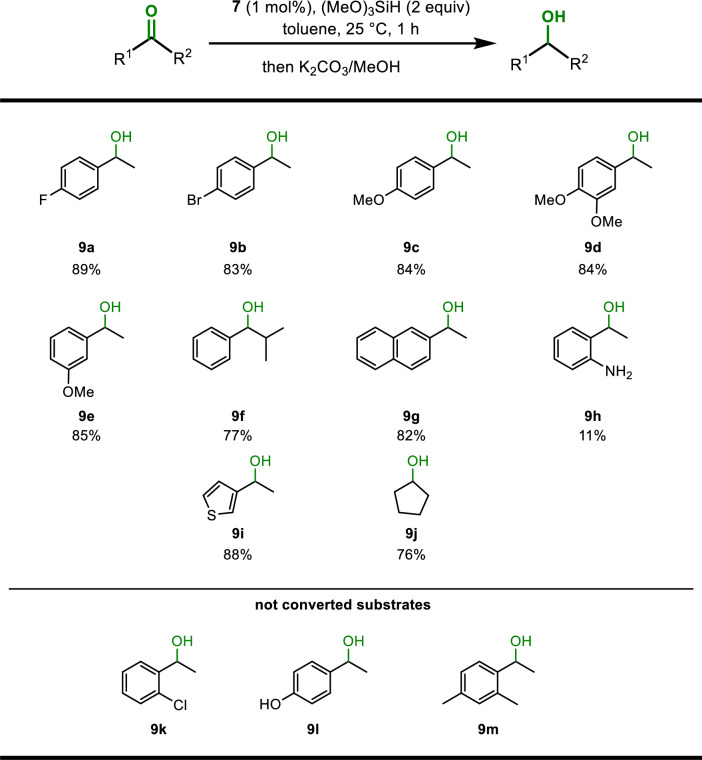
Hydrosilylation of ketones utilizing complex **7** as catalyst^a^

^a^Reaction conditions: 0.67 mmol ketone, 1.35 mmol Si(OMe)_3_H (2 equiv), 1 mol% catalyst, 3 cm^3^ toluene, 1 h, 25 °C. Isolated yields are reported

## Conclusion

In sum, we prepared and characterized several new Cr(II) and Cr(III) NCN pincer complexes with an arene backbone connected to amine donors NEt_2_ and N*i*Pr_2_ via CH_2_-linkers. The new complexes were typically prepared in a one-pot synthesis by reacting the in situ lithiated ligand precursors N(C–Br)N^CH2^-Et (**1**) and N(C–Br)N^CH2^-*i*Pr (**2**) with the Cr(III) and Cr(II) precursors [CrCl_3_(THF)_3_] and anhydrous Cr*X*_2_ (*X* = Cl, Br), respectively. In the case of Cr(III), complex *trans*-[Cr(κ^3^*NCN*-NCN^CH2^-Et)(Cl)_2_(THF)] (**3**) was obtained. In the case of Cr(II), the reaction of lithiated **2** with a suspension of anhydrous CrCl_2_, complex [Cr(κ^2^*NC*-NCN^CH2^-*i*Pr)_2_] (**4**) featuring two NCN ligands bound in κ^2^*NC*-fashion. If lithiated **2** is treated with a homogeneous solution of anhydrous Cr*X*_2_ (*X* = Cl, Br) complexes [Cr(κ^3^*NCN-*NCN^CH2^-*i*Pr)X] (**5a**, **5b**) are obtained where the NCN ligand is coordinated in the typical meridional κ^3^*NCN*-mode. Treatment of **5a** with 1 equiv. of PhCH_2_MgCl and LiCH_2_SiMe_3_ afforded the alkyl complex [Cr(κ^3^*NCN*-NCN^CH2^-*i*Pr)(CH_2_Ph)] (**6**) and [Cr(κ^3^*NCN*-NCN^CH2^-*i*Pr)-(CH_2_SiMe_3_)] (**7**), respectively. If a solution of lithiated **2** is treated with CrCl_2_ followed by the addition of an excess of Na[HB(Et)_3_] the dimeric complex [Cr(κ^2^*NC*-NCN^CH2^-*i*Pr)-(μ_2_-H)]_2_ (**8**) is formed bearing two µ_2_-hydride ligands bridging the two Cr(II) centers. This complex displays a solution magnetic moment of *μ*_eff_ = 6.9(3) *μ*_B_ which is consistent with eight unpaired electrons as expected for a high-spin d^4^ configuration of the two metal centers and no metal–metal interaction. This finding was also confirmed by DFT calculations. Finally, the alkyl complex [Cr(κ^3^*NCN*-NCN^CH2^-*i*Pr)(CH_2_SiMe_3_)] (**7**) turned out to be catalytically active for the hydrosilylation of aromatic and aliphatic ketones with Si(OMe)_3_H at room temperature with a catalyst loading of 1 mol%. X-ray structures of all complexes are presented.

## Experimental

All manipulations were performed under an inert atmosphere of argon by using Schlenk techniques or in an MBraun inert-gas glovebox. The solvents were purified according to standard procedures [32]. The deuterated solvents were purchased from Aldrich and dried over 3 Å molecular sieves. The ligands 1,1′-(2-bromo-1,3-phenylene)-bis-(*N*,*N*-diethylamine) (N(C–Br)N^CH2^-Et) (**1**) and 1,1′-(2-bromo-1,3-phenylene)-bis-(*N*,*N*-diisopropylamine) (N(C–Br)N^CH2^-iPr) (**2**) and anhydrous CrBr_2_ were synthesized according to literature [33–35]. All other materials are known compounds and were used as obtained from commercial suppliers. ^1^H, ^13^C{^1^H}, and ^19^F{^1^H} NMR spectra were recorded on Bruker AVANCE-250, AVANCE-400, and AVANCE-600 spectrometers. ^1^H and ^13^C{^1^H} NMR spectra were referenced internally to residual protio-solvent and solvent resonances, respectively, and are reported relative to tetramethylsilane (*δ* = 0 ppm). ^19^F{^1^H} NMR spectra were referenced externally to CFCl_3_.

High-resolution-accurate mass spectra were recorded on a hybrid Maxis Qq-aoTOF mass spectrometer (Bruker Daltonics, Bremen, Germany) fitted with an ESI-source. Measured accurate mass data of the [M]^+^ ions for confirming calculated elemental compositions were typically within 5 ppm accuracy. The mass calibration was done with a commercial mixture of perfluorinated trialkyl-triazines (ES Tuning Mix, Agilent Technologies, Santa Clara, CA, USA).

Electron Paramagnetic Resonance (EPR) spectra were recorded on an X-band Bruker Elexsys-II E500 CW-EPR spectrometer (Bruker Biospin GmbH, Rheinstetten, Germany) equipped with a high sensitivity cavity (SHQE1119) at 100 ± 1 K. The instrument parameters were set as follows: microwave frequency, 9.43 GHz; modulation frequency, 100 kHz, and microwave power, 15.9 mW. The spectra were analyzed using Xepr software and the Anisotropic SpinFit simulation program (both Bruker Biospin GmbH).

### [2,3-Bis[(diethylamino)methyl]phenyl-*C*,*N*,*N*′](dichloro)(tetrahydrofuran)chromium(III), [Cr(κ^3^*NCN*-NCN^CH2^-Et)(Cl)_2_(THF)] (3, C_20_H_35_Cl_2_CrN_2_O)

A solution of (N(C–Br)N^CH2^-Et) (**1**, 400 mg, 1.23 mmol) in THF (13 cm^3^) was cooled to − 90 °C. After stirring for 10 min, *n*-BuLi (1.52 cm^3^, 1.6 M in hexanes, 2.46 mmol) was added dropwise via a syringe and the light-yellow solution was stirred at − 90 °C for 2.5 h. After warming up to − 30 °C, [CrCl_3_(THF)_3_] (460 mg, 1.23 mmol) was added portion-wise under stirring resulting in the formation of a dark green solution. The solution was allowed to warm to room temperature and was stirred for 12 h. After removal of the solvent under reduced pressure, the residue dissolved in toluene (7 cm^3^) and filtered through celite. After removal of the toluene, the residue was washed three times with *n*-pentane (3 × 10 cm^3^). Drying under reduced pressure yielded a light-purple solid. Yield: 340 mg (63%). Dark green crystals suitable for X-ray crystallography were obtained by slow diffusion of *n*-pentane into a saturated THF solution of **3**. *µ*_eff_ = 3.7(2) *µ*_B_ (Evans method, THF); HR-MS (ESI^+^, THF): *m/z* calcd for C_16_H_27_Cl_2_CrN_2_ ([M-THF+H]^+^) 370.1029, found 370.1031.

### Bis-[2,3-bis[(diisopropylamino)methyl]phenyl-***C***,***N***,***N***′]-chromium(II), [Cr(κ^2^***NC***-NCN^CH2^-***i***Pr)_2_] (4, C_40_H_70_CrN_4_)

A solution of (N(C–Br)N^CH2^-*i*Pr) (**2**, 100 mg, 0.26 mmol) in THF (7 cm^3^) was cooled to − 90 °C. After stirring at that temperature for 10 min, *n*-BuLi (0.18 cm^3^, 1.6 M, 0.30 mmol) was added dropwise via a syringe. A suspension of anhydrous CrCl_2_ (38 mg, 0.30 mmol) in THF (4 cm^3^) was added in a dropwise fashion at − 20 °C. After the addition was completed, all volatiles were removed under reduced pressure and the residue was extracted with *n*-pentane (6 cm^3^) and filtered through a syringe filter (PTFE, 0.2 µm). The solvent was removed under vacuum yielding a dark blue solid. Yield: 45 mg (37%). Blue crystals suitable for X-ray crystallography were obtained by cooling a saturated *n*-pentane solution of **4** to − 30 °C. *µ*_eff_ = 4.7(3) *µ*_B_ (Evans method, benzene). HR-MS spectra could not be obtained due to the highly air-sensitive nature of the compound.

### [2,3-Bis[(diisopropylamino)methyl]phenyl-***C***,***N***,***N***′]-(chloro)chromium(II), [Cr(κ^3^***NCN-***NCN^CH2^-***i***Pr)Cl] (5a, C_20_H_35_ClCrN_2_)

Anhydrous CrCl_2_ (38 mg, 0.30 mmol) was suspended in THF (15 cm^3^). The suspension was exposed to ultrasonic irradiation for 1 h to form a light-blue clear solution. The ligand (N(C–Br)N^CH2^-*i*Pr) (**2**) (100 mg, 0.26 mmol) was dissolved in THF (5 cm^3^) and subsequently cooled to − 90 °C. After stirring at that temperature for 10 min, *n*-BuLi (0.18 cm^3^, 1.6 M, 0.30 mmol) was added dropwise via a syringe. The orange solution was stirred for 30 min before it was allowed to warm to 0° C while stirring for another hour. The lithiated intermediate was added in a dropwise fashion to the THF solution of CrCl_2_ via a syringe. The solution was allowed to stir for 15 min at room temperature before all volatiles were evaporated under vacuum. The residue was extracted into *n*-pentane (7 cm^3^) and filtered through a syringe filter (PTFE, 0.2 µm). The volume of the filtrate was reduced to about 1.5 cm^3^ and stored at − 30 °C to afford **5** as dark purple crystalline plates suitable for X-ray diffraction analysis. Yield: 75 mg (73%); *µ*_eff_ = 4.8(2) *µ*_B_ (Evans method, benzene). HR-MS spectra could not be achieved due to the highly air-sensitive nature of the compound.

### [2,3-Bis[(diisopropylamino)methyl]phenyl-***C***,***N***,***N***′]-(bromo)chromium(II), [Cr(κ^3^***NCN-***NCN^CH2^-***i***Pr)Br (5b, C_20_H_35_BrCrN_2_)

Complex **5b** was prepared analogously to complex **5a** utilizing anhydrous CrBr_2_ (58 mg, 0.30 mmol) and N(C–Br)N^CH2^-iPr (**2**) (100 mg, 0.26 mmol) as starting materials. Yield: 85.2 mg (75%); *µ*_eff_ = 4.7(2) *µ*_B_ (Evans method, benzene). HR-MS spectra could not be obtained due to the highly air-sensitive nature of the compound.

### [2,3-Bis[(diisopropylamino)methyl]phenyl-*C*,*N*,*N*′](phenyl)-(methyl)) chromium(II), [Cr(κ^3^*NCN*-NCN^CH2^-*i*Pr)(CH_2_Ph)](6, C_27_H_42_CrN_2_)

*Method A.* A suspension of anhydrous CrCl_2_ (38 mg, 0.30 mmol) in THF (15 cm^3^) was placed in an ultrasonic bath for 1 h whereupon the suspension turned into light blue solution. (N(C–Br)N^CH2^-*i*Pr) (**2**, 100 mg, 0.26 mmol) was dissolved in THF (5 cm^3^) and subsequently cooled to − 90 °C. After stirring for 10 min, *n*-BuLi (0.18 cm^3^, 1.6 M, 0.30 mmol) was added dropwise and the solution was stirred for additional 30 min. The solution was allowed to warm to 0 °C and was stirred for 1 h. The lithiated intermediate was then added in a dropwise fashion to THF solution of CrCl_2_ and a dark-colored solution was formed immediately. The solution was stirred for 15 min at room temperature and all volatiles were then evaporated under vacuum. The residue was dissolved in benzene and the solution was filtered through a syringe filter (PTFE, 0.2 µm). A solution of PhCH_2_MgCl (0.1 cm^3^, 1 M in THF) was then added and the suspension was stirred for 1.5 h. All volatiles were removed under reduced pressure. The remaining residue was extracted with *n*-pentane (3 cm^3^) and filtered through syringe filter (PTFE, 0.2 µm). Evaporation of the solvent afforded **6** as a dark brown solid. Cooling of a saturated *n*-pentane solution of 6 to − 30° C yielded crystals suitable for X-ray diffraction. Yield: 81 mg (72%); *µ*_eff_ = 4.9(1) *µ*_B_ (Evans method, benzene).

*Method B.* Complex **6** was also obtained by reacting isolated **5a** (50 mg, 0.12 mmol) in benzene (2 cm^3^) with PhCH_2_MgCl (0.14 cm^3^, 1 M in THF) and stirring for 1.5 h at room temperature. After evaporating the solvent under reduced pressure, redissolving the residue in* n*-pentane (2 cm^3^) and subsequent filtration (syringe filter PTFE, 0.2 µm) and removal of the solvent, **6** could be isolated as a dark brown solid. Yield: 41 mg (72%). HR-MS spectra could not be achieved due to the highly air-sensitive nature of the compound.

### [2,3-Bis[(diisopropylamino)methyl]phenyl-*C*,*N*,*N*′](trimethylsilane)(methyl)chromium(II), [Cr(κ^3^*NCN*-NCN^CH2^-*i*Pr)-(CH_2_SiMe_3_)] (7, C_24_H_46_CrN_2_Si)

*Method A.* Complex **7** was prepared in an analogous fashion to **6** utilizing LiCH_2_TMS (0.28 cm^3^, 0.28 mmol, 1 M in *n*-pentane). Complex **7** was obtained as a dark brown solid. Yield: 76 mg (68%); *µ*_eff_ = 4.8(1) µ_B_ (Evans method, benzene).

*Method B.* A solution of Complex **5a** (50 mg, 0.12 mmol) in benzene (2 cm^3^) was treated with LiCH_2_TMS (0.13 cm^3^, 0.28 mmol, 1 M in *n*-pentane) and stirring for 1.5 h at room temperature. After evaporation of the solvent, redissolving the residue in* n*-pentane (2 cm^3^) and subsequent filtration (syringe filter PTFE, 0.2 µm) and removal of the solvent, **7** could be isolated as a dark brown solid. Yield: 39 mg (69%). HR-MS spectra could not be obtained due to the highly air-sensitive nature of the compound.

### Bis-[2,3-bis[(diisopropylamino)methyl]phenyl-*C*,*N*,*N*′])-(μ_2_-hydrido)chromium(II)], [Cr(κ^2^*NC*-NCN^CH2^-*i*Pr)(μ_2_-H)]_2_ (8, C_40_H_72_Cr_2_N_4_)

A suspension of anhydrous CrCl_2_ (38 mg, 0.30 mmol) in THF (15 cm^3^) was placed in an ultrasonic bath for 1 h, whereupon a light blue solution was obtained. The ligand precursor N(C–Br)N^CH2^-*i*Pr (**2**, 100 mg, 0.26 mmol) was dissolved in THF (5 cm^3^) and cooled to − 90 °C. After stirring for 10 min, *n*-BuLi (0.18 cm^3^, 1.6 M, 0.30 mmol) was added dropwise via a syringe and the orange solution was further stirred for 30 min and then allowed to warm to 0 °C and stirred for an additional 1 h. The solution containing lithiated N(C–Br)N^CH2^-*i*Pr (**2**) was added dropwise to the THF-solution of anhydrous CrCl_2_ whereupon the solution became dark purple. The solution was allowed to stir for 15 min at room temperature before all volatiles were evaporated under vacuum. The residue was dissolved in benzene and filtered through a syringe filter (PTFE, 0.2 µm) and volatiles were evaporated under reduced pressure. The residue was dissolved in precooled THF (− 30 °C, 3 cm^3^) and treated with NaHBEt_3_ (0.91 cm^3^, 0.91 mmol, 1 M in THF) and stirred for 30 min. After removal of the solvent, the remaining residue was redissolved in *n*-pentane (10 cm^3^), filtered through a syringe filter (PTFE, 0.2 µm) and the volume of the solution was reduced to approximately 1 cm^3^. Storage of the light brown solution at -30 °C for 12 h led to the formation of bright orange crystals. Yield: 64 mg (35%); *µ*_eff_ = 6.9(3) µ_B_ (Evans method, benzene-*d*_*6*_); HR-MS (ESI^+^, THF): *m/z* calcd for C_40_H_72_Cr_2_N_4_ ([M-C_20_H_36_CrN_2_]^+^) 356.2279, found 356.2278.

### General procedure for the hydrosilylation of ketones

To a solution of the substrate (0.67 mmol, 1 equiv.) and HSi(OMe)_3_ (174 mm^3^, 1.35 mmol, 2 equiv) in toluene (3 cm^3^) complex **7** was added (3 mg, 6.7 µmol, 1 mol%) causing an immediate color change. The mixture was stirred at room temperature for 1 h and then a saturated methanolic K_2_CO_3_ solution (3 cm^3^) was added to the mixture. After stirring for 5 h volatiles were evaporated under reduced pressure. The residue was dissolved in CH_2_Cl_2_ (6 cm^3^) and filtered through a short plug of silica. After evaporation of the solvent, the reaction products were analyzed by the means of ^1^H, ^13^C {^1^H}, and ^19^F{^1^H} NMR spectroscopy.

### X-ray structure determination

X-ray diffraction data of **3**, **4**, **5a** (CCDC 2285377–2285379) and **8** (CCDC 2285381) were collected at *T* = 100 K in a dry stream of nitrogen on a Bruker Kappa APEX II diffractometer system using graphite-monochromatized Mo-*K*α radiation (*λ* = 0.71073 Å) and fine sliced *φ*- and *ω*-scans. Data were reduced to intensity values with SAINT and an absorption correction was applied with the multi-scan approach implemented in SADABS [36]. Data of **6** (CCDC 2285380) were collected at *T* = 100 K on a Rigaku XtaLAB Synergy, Dualflex diffractometer system equipped with a HyPix hybrid photon counting detector using Cu-*K*α radiation (*λ* = 1.54184 Å). Data were reduced and an absorption correction applied using the multi-scan approach with the CrysAlisPro software [37]. The structures were solved by the dual-space approach implemented in SHELXT [38] and refined against *F*^2^ with SHELXL [39]. Non-hydrogen atoms were refined with anisotropic displacement parameters. H atoms attached to C were placed in calculated positions and thereafter refined as riding on the parent atoms. The positions of the hydride hydrogens in **8** were freely refined. The halogenide ligands in **3** and **5a** were modelled as occupationally disordered Cl/Br sites with the total occupation of each sit e constrained to 1. Molecular graphics were generated with the program MERCURY [40].

## Computational details

The computational results presented have been achieved in part using the Vienna Scientific Cluster (VSC). Calculations were performed using the GAUSSIAN 09 software package [41] and the OPBE [42–45] functional without symmetry constraints, the Stuttgart/Dresden ECP (SDD) basis set [46–48] to describe the electrons of the chromium atom and a standard 6-31G** basis for all other atoms as already previously described [49–53]. Population analysis (NPA) [54–61] and the resulting Wiberg indices [31] were used to study the electronic structure and bonding of the optimized species. The NPA analysis was performed with the NBO 5.0 program [62]. The α-spin orbitals and spin density drawings (Fig. 7) were obtained with the program Molekel [63].

### Supplementary Information

Below is the link to the electronic supplementary material.

Supplementary file1 (PDF 105 KB)

Supplementary file2 (CIF 4940 KB)

Supplementary file3 (XYZ 9 KB)

## Data Availability

All relevant data are included in the manuscript.

## Footnotes

1 Wiberg indices are electronic parameters related with the electron density in between two atoms, which scale as bond strength indicators. They can be obtained from a Natural Population Analysis.
