# Supplementary material for: Cr(II) and Cr(III) NCN pincer complexes: synthesis, structure, and catalytic reactivity
Source: Monatsh Chem. 2023 Oct 10;154(11):1263–73. doi: 10.1007/s00706-023-03128-6 (PMC10620270; doi:10.1007/s00706-023-03128-6)
Supplement: Supplementary file 1 — Supplementary file1 (PDF 105 KB) [file 706_2023_3128_MOESM1_ESM.pdf]

Cr(II) and Cr(III) NCN Pincer Complexes: Synthesis, Structure, and Catalytic Reactivity

Matthias G. Käfer[1] ● Wolfgang Eder[1] ● Jan Pecak[1] ● Berthold Stöger[2+] ● Marc Pignitter[3] ● Luis F. Veiros[4] ● Karl Kirchner[1,*]

[1] Institute of Applied Synthetic Chemistry, TU Wien, Getreidemarkt 9/163-AC, 1060 Wien, Austria.

[2] X-Ray Center, TU Wien, Getreidemarkt 9/163-AC, 1060 Wien, Austria.

[3] Department of Physiological Chemistry, Faculty of Chemistry, University of Vienna, Althanstraße 14, 1090 Vienna, AUSTRIA

[4] Centro de Química Estrutural, Institute of Molecular Sciences, Departamento de Engenharia Química, Instituto Superior Técnico, Universidade de Lisboa, Av. Rovisco Pais, 1049 001 Lisboa, Portugal

Supporting Information

EXPERIMENTAL

Hydrosilylation of Ketones

Optimization of reaction conditions at 25 ºC, using complex 7 as a catalyst.

entry	Silane	equiv. Silane	Cat. [mol%]	t [h]	Solvent	Conversion [%]
**1**	$(MeO)_3SiH$	2	5	2	C_6D_6	>99
**2**	$(MeO)_3SiH$	2	5	2	C_6D_6	>99
**3**	$(MeO)_3SiH$	2	3	2	C_6D_6	>99
**4**	$(MeO)_3SiH$	2	1	2	C_6D_6	>99
**5**	$(MeO)_3SiH$	2	1	1	C_6D_6	>99
**6**	$(MeO)_3SiH$	2	1	1	toluene	>99
**7**	$(MeO)_3SiH$	1.5	1	2h	pentane	45
**8**	$(MeO)_3SiH$	1.5	0.5	2h	pentane	27
**9**	$PhMe_2SiH$	2	1	0.	toluene	0
**10**	$PhSiH_3$	2	1	2	toluene	0
**11**	$(MeO)_3SiH$	1.5	1	1	toluene	87
**12**	$HSiEt_3$	2	1	2	Toluene	0

Substrate Scope and Limitations

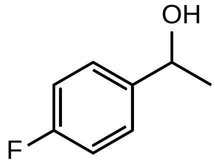

1-(4-fluorophenyl)ethanol (9a)

Yield: 84.5 mg (89%)

^{1}H NMR (400 MHz, $CDCl_3$, 20 °C, ppm): δ 7.40 – 7.30 (m, 2H), 7.10 – 6.99 (m, 2H), 4.89 (dp, *J* = 6.4, 3.2 Hz, 1H), 1.49 (d, *J* = 6.4 Hz, 3H).

^{19}F{^{1}H} NMR (376 MHz, $CDCl_3$, 20 °C, ppm) δ -115.34.
^{13}C NMR (δ, 101 MHz, $CDCl_3$, 20 °C, ppm): 162.2 (d, JCF = 243.1 Hz), 141.9, 127.1, 115.3 (d, JCF = 21.1 Hz), 69.8, 25.6
Reported Data corresponds to literature data [1]

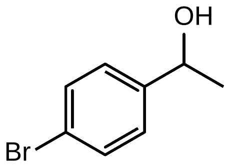

1-(4-bromophenyl)ethanol (9b)

Yield: 113.1 mg (83%)

^{1}H NMR (400 MHz, $CDCl_3$, 20 °C, ppm): δ 7.47 (d, *J* = 8.4 Hz, 2H), 7.27 (d, *J* = 0.6 Hz, 1H), 7.25 – 7.24 (m, 1H), 4.92 – 4.84 (m, 1H), 1.78 (t, *J* = 5.3 Hz, 1H), 1.48 (d, *J* = 6.5 Hz, 3H).

^{13}C NMR (δ, 101 MHz, $CDCl_3$, 20 °C, ppm): δ 144.4, 131.7, 127.3, 121.4, 69.1, 25.2
Reported Data corresponds to literature data [2]

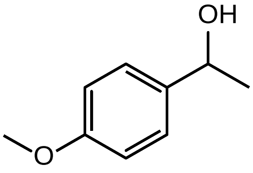

1-(4-methoxyphenyl)ethanol (9c)

Yield:86.6 mg (84 %)

^{1}H NMR (400 MHz, CDCl$_3$, 20 °C, ppm): δ 7.35 – 7.28 (m, 2H), 6.91 – 6.87 (m, 2H), 4.86 (qd, *J* = 6.4, 3.3 Hz, 1H), 3.80 (s, 3H), 1.48 (d, *J* = 6.4 Hz, 3H)

^{13}C NMR (101 MHz, CDCl$_3$, 20°C, ppm): δ 159.1, 138.2, 126.8, 113.9, 70.0, 55.4, 25.2..
Reported Data corresponds to literature data [3]

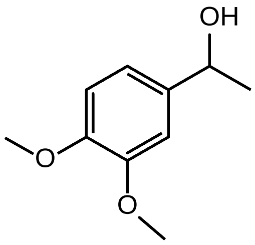

1-(3,4-dimethoxyphenyl)ethanol (9d)

Yield: 104.9 mg (85%)

^{1}H NMR (400 MHz, CDCl$_3$, 20 °C, ppm): δ 6.89 (t, *J* = 1.7 Hz, 1H), 6.86 – 6.82 (m, 2H), 4.86 (qd, *J* = 6.4, 3.4 Hz, 1H), 3.90 (s, 3H), 3.88 (s, 3H), 1.74 (d, *J* = 3.4 Hz, 1H), 1.50 (d, *J* = 6.4 Hz, 3H).

^{13}C NMR (δ, 101 MHz, CDCl$_3$, 20 °C, ppm): δ 149.2, 148.6, 138.4, 117.5, 111.3, 108.2, 70.3, 56.1, 55.4, 25.1
Reported Data corresponds to literature data [4]

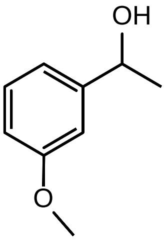

1-(3-methoxyphenyl)ethanol (9e)

Yield: 87.60 mg (85%)

^{1}H NMR (400 MHz, CDCl$_3$, 20 °C, ppm): δ 7.19 (t, *J* = 8.3 Hz, 1H), 6.98 – 6.93 (m, 2H), 6.82 (ddd, *J* = 8.2, 2.6, 1.0 Hz, 1H), 4.94 – 4.83 (m, 1H), 3.82 (d, *J* = 0.7 Hz, 3H), 1.77 (d, *J* = 3.6 Hz, 1H), 1.50 (d, *J* = 6.5 Hz, 3H).

^{13}C NMR (101 MHz, CDCl$_3$, 20°C, ppm): δ 159.2, 147.7, 129.5, 116.7, 113.6, 111.2, 71.2, 55.2, 25.2
Reported Data corresponds to literature data.[1]

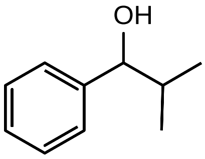

2-methyl-1-phenylpropan-1-ol (9f)

Yield: 78.3 mg (77%)

^{1}H NMR (400 MHz, CDCl$_3$, 20 °C, ppm): δ 7.22 – 7.14 (m, 5H), 4.60 – 4.50 (m, 1H), 2.03 – 1.82 (m, 2H, CH +OH), 0.98 (d, *J* = 6.7 Hz, 3H), 0.80 (d, *J* = 6.8 Hz, 3H).

^{13}C NMR (101 MHz, CDCl$_3$, 20°C, ppm): δ 143.7, 128.1, 127.2, 126.9, 80.3, 35.1, 19.4, 18.2
Reported Data corresponds to literature data. [2]

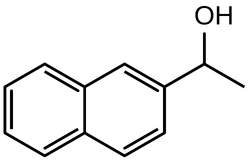

1-(naphthalen-2-yl)ethanol (9g)

Yield: 95.6 mg (82%)

^{1}H NMR (400 MHz, CDCl$_3$, 20 °C, ppm): δ 7.88 – 7.81 (m, 4H), 7.54 – 7.41 (m, 3H), 5.08 (tt, *J* = 6.5, 3.2 Hz, 1H), 1.59 (d, *J* = 6.5 Hz, 3H).

^{13}C NMR (101 MHz, $CDCl_3$, 20°C, ppm): 144.0, 133.7, 133.2, 128.4. 128.3, 128.0, 126.4, 126.1, 124.4, 124.1, 70.6, 25.4.
Reported Data corresponds to literature data [5]

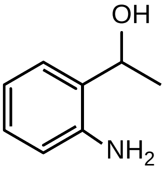

<u>1-(2-aminophenyl)ethanol (9h)</u>

<u>Yield:</u> 10.2 mg (11 %)

^{1}H NMR (400 MHz, $CDCl_3$, 20 °C, ppm): δ 7.06 (ddt, *J* = 13.6, 11.9, 8.3 Hz, 4H), 5.13 (q, *J* = 6.6 Hz, 1H), 4.20 (s, 4H), 1.61 (d, *J* = 6.6 Hz, 3H).

^{13}C NMR (101 MHz, $CDCl_3$, 20°C, ppm): 145.2, 128.6, 126.6, 118.4, 116.8, 69.7, 22.1
Reported Data corresponds to literature data. [4]

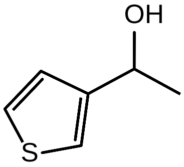

<u>1-(thiophen-3-yl)ethanol (9i)</u>

<u>Yield:</u> 76.4 mg (88 %)

^{1}H NMR (400 MHz, $CDCl_3$, 20 °C, ppm): δ 7.31 (dd, *J* = 5.0, 3.0 Hz, 1H), 7.21 – 7.18 (m, 1H), 7.11 (dd, *J* = 5.1, 1.3 Hz, 1H), 5.03 – 4.93 (m, 1H), 1.53 (d, *J* = 6.4 Hz, 3H).

^{13}C NMR (101 MHz, $CDCl_3$, 20°C, ppm): 150.0, 126.5, 124.1, 122.9, 66.3, 25.3
Reported Data corresponds to literature data [6]

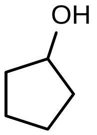

<u>Cyclopentanol (9j)</u>

<u>Yield:</u> 44.3 mg (76%)

^{1}H NMR (400 MHz, $CDCl_3$, 20 °C, ppm): δ 4.53 – 4.45 (m, 1H), 1.79 – 1.66 (m, 4H), 1.62 – 1.47 (m, 4H)
(O*H* not detected)

^{13}C NMR (101 MHz, $CDCl_3$, 20°C, ppm): δ 74.3, 35.4, 23.4
Reported Data corresponds to literature data [5]

NOT CONVERTED SUBSTRATES

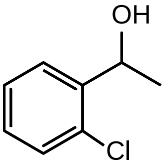

<u>1-(2-chlorophenyl) ethanol (9k)</u>

Yield: *no product formation observed*

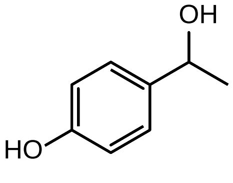

<u>1-(4-hydroxyphenyl) ethanol (9l)</u>

Yield: *no product formation observed*

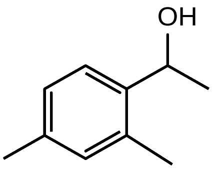

<u>1-(2,4-dimethylphenyl) ethanol (9m)</u>

Yield: *no product formation observed*

REFERENCES

1. Li L, Cheng J, Zhang G (2018) Dalton Trans 47:9575
2. Wie D, Bruneaz-Voisine A, Chauvin T, Dorcet V, Roisinel T, Valyaev DA, Lugan N, Sortais JB (2018) Adv Synth Catal 360:676
3. Ganglui K, Shee S, Panja D, Kundu S, (2019) Dalton Trans 48:7358
4. Zhu K, Shaver MP, Thomas SP (2015) Eur J Org Chem 10:2119
5. Himmelbauer D, Stöger B, Veiros LF, Pignitter M, Kirchner K (2019) Organometallics 38:4669
6. Xuan Q, Zhao C, Song Q (2017) Org Biomol Chem 15:5140
7. Kurogi T, Irifune K, Takai K (2021) Chem Sci 12:14281
